# Mechanisms mediating the impact of maternal obesity on offspring hypothalamic development and later function

**DOI:** 10.3389/fendo.2022.1078955

**Published:** 2022-12-22

**Authors:** Isadora C. Furigo, Laura Dearden

**Affiliations:** ^1^ Centre for Sport, Exercise and Life Sciences, School of Life Sciences, Coventry University, Coventry, United Kingdom; ^2^ Metabolic Research Laboratories, Wellcome MRC Institute of Metabolic Science, University of Cambridge, Cambridge, United Kingdom

**Keywords:** Obesity, hypothalamus, pregnancy, developmental programming, food intake

## Abstract

As obesity rates have risen around the world, so to have pregnancies complicated by maternal obesity. Obesity during pregnancy is not only associated with negative health outcomes for the mother and the baby during pregnancy and birth, there is also strong evidence that exposure to maternal obesity causes an increased risk to develop obesity, diabetes and cardiovascular disease later in life. Animal models have demonstrated that increased weight gain in offspring exposed to maternal obesity is usually preceded by increased food intake, implicating altered neuronal control of food intake as a likely area of change. The hypothalamus is the primary site in the brain for maintaining energy homeostasis, which it coordinates by sensing whole body nutrient status and appropriately adjusting parameters including food intake. The development of the hypothalamus is plastic and regulated by metabolic hormones such as leptin, ghrelin and insulin, making it vulnerable to disruption in an obese *in utero* environment. This review will summarise how the hypothalamus develops, how maternal obesity impacts on structure and function of the hypothalamus in the offspring, and the factors that are altered in an obese *in utero* environment that may mediate the permanent changes to hypothalamic function in exposed individuals.

## 1 Introduction

There can be no doubt that the world is in the midst of an obesity crisis. The rise in obesity has occurred in both sexes and across all ages, meaning that there has inevitably been an increase in the number of pregnancies complicated by obesity. The most recent figures from the UK collected in 2019 show that 27.4% of women were overweight and 21.6% were living with obesity or severe obesity at the time of their first antenatal appointment (usually around 8-10 weeks gestation) (1). In the USA, figures from 2019 show that 29% of women were living with obesity when they became pregnant (2). This data is from before the Covid-19 pandemic, during which we know that obesity rates have risen globally. Post- pandemic figures from Scotland collected in 2021 show that 25.9% of women entered pregnancy whilst living with obesity or severe obesity (3).

Obesity during pregnancy is associated with negative health outcomes for the mother and the baby both during pregnancy and birth. For the mother, obesity is associated with greater odds of developing gestational diabetes (GDM), hypertension and the life-threatening condition pre-eclampsia. For the fetus, maternal obesity is associated with an increased risk of stillbirth, being born both small or large for gestational age, and an increased incidence of emergency caesarean birth. As well as these immediate effects on the health of the mother and the baby, exposure to maternal obesity during pregnancy is also associated with more long-term health problems in offspring. There is now strong evidence that exposure to maternal obesity causes an increased risk to develop obesity, diabetes, and cardiovascular disease later in life.

It is extremely concerning that the most recent report from the National Child Measurement Programme in the UK reported that nearly 30% of children in Reception (age 4 and 5 years old) are overweight or living with obesity (4). The children of today are the parents of tomorrow- meaning that pregnancies complicated by parental obesity are likely to become even more common. It is clear there is a pressing need for more interventions- be they lifestyle based or pharmacological- to stop the inter-generational transmission of obesity risk. There is also a lack of sound advice for expectant parents- in many cultures the old missive of “eating for two” during pregnancy still prevails. However, in order for researchers and health care professionals to deliver sound clinical advice and interventions, we first need to understand the mechanisms by which changes in the *in utero* environment of an obese pregnancy are translated into an increased cardio- metabolic disease risk in offspring. Animal models have consistently demonstrated that increased weight gain in offspring exposed to maternal obesity is preceded by increase food intake, implicating altered neuronal control of food intake as a likely area of change. The hypothalamus is the primary site in the brain for maintaining energy homeostasis, which it does by appropriately adjusting parameters including food intake. This review will summarise how the hypothalamus develops, how maternal obesity impacts on structure and function of the hypothalamus, and the factors that are altered in an obese *in utero* environment that may program these changes.

## 2 Development of hypothalamic energy balance circuits

Neurons and regions within the neuroendocrine portion of the hypothalamus are largely characterized by the neuropeptides and neurotransmitters that have been defined over the last half-century. As researchers continue to uncover the complexity of hypothalamic circuitry it is becoming clear that it may not be sufficient to classify a neuronal sub-type based on its expression of a specific, well-known neuropeptide. Indeed, recent studies have demonstrated the heterogeneity of neurons not only within a defined region of the hypothalamus, such as the arcuate nucleus (ARC) (5) but also within what was previously thought of as being one sub-type of neuron, for example proopiomelanocortin (POMC) neurons (6). Continued efforts to define the genetic make-up of all neurons within the hypothalamus and collate this into large datasets (7) will undoubtedly further our understanding of how the hypothalamus develops, as well as functions.

The data described in this section is essentially limited to rodents, as- for obvious reasons- there is a paucity of data on hypothalamic development in non-human primates (NHP) and humans. Although the highly conserved functions of hypothalamic regions between rodents and higher organisms suggest that many developmental mechanisms may be shared, our knowledge of NHP and human hypothalamic development is far from complete. The hypothalamus is a region of the brain where neurons are added for an extended prenatal period and are even born postnatally (8). There is also considerable remodelling of hypothalamic connections during the early post-natal period. Therefore, both the fetal and neonatal period represent critical periods of vulnerability in the hypothalamus.

### 2.1 Neurogenesis and cell fate determination

Over the past fifty years, numerous models have been proposed as to how the forebrain, including the hypothalamus, develops. Although many early models have now been disproved by the advent of data showing the temporal expression of specific transcription factors, there remains a dispute as to whether the hypothalamus and telencephalon should be classed as a single unit, termed the secondary prosencephalon (9), or whether the hypothalamus is part of the diencephalon (10). The cells that will form the hypothalamus are primarily derived from precursor cells located in a proliferative zone in the neuroepithelium of the third ventricle. In rodents, by embryonic day (E) 10, the presumptive hypothalamus has acquired regional identity by the combined action of morphogens such as Shh (extensively reviewed in (11)) and a well-characterised transcriptional network (e.g., *Nkx2.1, Six3, Otp*). For an excellent comprehensive review of early hypothalamic regional patterning and progenitor cell determination see Burbridge et al. (12). The early hypothalamus can be roughly divided into three regions along it’s rostral to caudal axis- the anterior, tuberal and posterior regions. Once these hypothalamic regions are established, the progenitors within begin to acquire their subtype-specific identities.

The neuroendocrine portion of the hypothalamus- which is the primary site of feeding regulatory pathways- comprises the anterior and tuberal hypothalamus. In the tuberal hypothalamus, all neurons are produced during a period of only a few days (13). The majority of neurons in the paraventricular nucleus (PVH) and dorsomedial nucleus (DMH) are generated between E12-E14, whereas the ARC and ventromedial nucleus (VMH) have longer periods of neuronal generation from E12-E16 (14, 15). Following this initial wave of neurogenesis in the hypothalamus, there is a gradual shift to gliogenesis that generates hypothalamic astrocytes (13).

The Notch signalling pathway is a key regulator of neurogenesis in the central nervous system (CNS). As Notch signalling inhibits pro-neural genes, models lacking Notch signalling exhibit an increase in neurons throughout the embryo (16). Conditional loss of function mice using *Nkx2.1*-Cre to specifically knock out Notch signalling in the hypothalamus show that Notch signalling is essential for the differentiation of late ARC neurons in the mouse from E13.5 (17). The pro-neural transcription factor Mash1 is inhibited by Notch signalling, and loss of Mash1 in a mouse model is associated with a reduction of POMC and NPY neurons in the ARC (18). Consistent with this study, mice lacking Notch signalling in hypothalamic cells show an increased number of POMC and NPY neurons (17).

### 2.2 Neurite extension

Neuronal axons grow by extending a growth cone, which travels toward a target and trails behind it the growing neurite. The path of a growing axon is determined by cell-cell interactions and diffusible chemorepulsive and chemoattractive cues. Axon guidance molecules such as netrins (19), ephrins (20) and semaphorins have been show to regulate axon growth in the hypothalamus. Semaphorins are one of the largest family of guidance molecules. The semaphorin 3 family members are required for correct development of hypothalamic reproductive pathways (21), and the melanocortin system; if the semaphorin receptor Neuropillin is disrupted then neurite projections from the ARC innervate the VMH rather than their correct target, the PVH (22).

Dii tracing studies demonstrate that hypothalamic connections develop with a high degree of spatial and temporal specificity, innervating each target with a unique developmental schedule which in many cases can be correlated with the functional maturity of the projection (23). The development of axonal projections from the ARC begins prenatally. As early as E14, there are long descending projections from POMC neurons that follow a longitudinal route towards the upper thoracic spinal cord (24). Intra-hypothalamic connections from the ARC to other hypothalamic areas such as the PVH form post-natally. Studies by Bouret et al. have elegantly demonstrated that projections from the ARC do not represent an adult distribution until post-natal day (PND) 18 in mice, with connections specifically between the ARC and PVH forming between PND8-10 (25). Further studies in rodents have demonstrated that orexigenic NPY positive neurons from the ARC innervate the PVH at PND10-11, but brainstem NPY positive neuronal fibers arrive at the PVH much earlier and are present from PND2 (26). In comparison, in the NHP the development of NPY positive projections from the ARC occurs during the third trimester of gestation, and offspring are born with an abundance of NPY positive fibers originating from the ARC. However, the pattern of ARC projections seen in the NHP in late gestation is less dense than in adults, suggesting additional refinement of the connectivity occurs in the post-natal period (27), as in rodents.

### 2.3 Epigenetic regulation of neural maturation

It is becoming increasingly apparent that epigenetic processes play an important role in maturation of the hypothalamus. Extensive changes in DNA methylation that differentiate between neurons and non-neuronal sub types in the ARC and PVH occur in the early post-natal period in rodents (28). The activity of both methylating and de-methylating enzymes in the hypothalamus is developmentally regulated and shows different activity patterns between male and female brains (29). Most recently, MacKay et al. have shown that post-natal epigenetic maturation in the ARC is cell type and sex specific and occurs particularly in genomic regions enriched for heritability of BMI in humans (30).

There is accumulating evidence that miRNAs, which are small non- coding RNAs that regulate gene function through degradation of mRNAs and/or inhibition of protein translation (31), may reflect an important mechanism by which maternal environment can alter long-term phenotypes in the offspring (32, 33). Although hypothalamic miRNAs are still not well characterised- either in adult life or during development- a recent paper has shown that 30% of the miRNAs present in the ARC show altered expression throughout the post-natal period (34). Furthermore, Croizier and colleagues have shown that miR103/107 are required for the process when POMC expressing precursors switch to become mature NPY expressing neurons (35).

## 3 Impact of maternal obesity on the offspring hypothalamus

**Figure 1:**
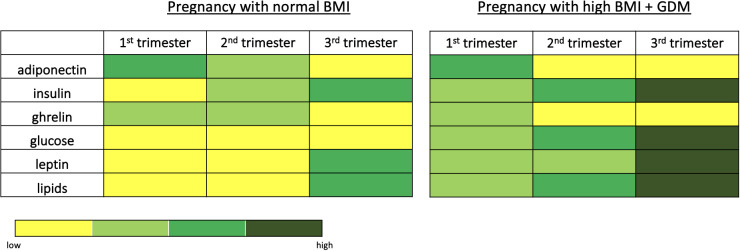
Main routes by which maternal obesity impacts on hypothalamic development and function in the offspring. Animal models have shown that exposure to maternal obesity impacts on hypothalamic development throughout fetal development. Maternal obesity is associated with reduced proliferation of hypothalamic progenitor cells in the fetal and neonatal hypothalamus, whether this is a permanent reduction or a delay in the normal neurogenic process is unknown. The formation of intra- hypothalamic projections- particularly in the melanocortin system- is also disrupted in offspring exposed to maternal obesity. This may be due to altered signalling of neurotrophic factors such as leptin, resulting in a reduction in neurite projections, or altered expression of axon guidance cues, resulting in incorrect targets of growing neurites. The offspring of obese mothers also show reduced nutrient sensing, which is one of the primary functions of the hypothalamus and required for the correct regulation of energy homeostasis.

The hypothalamus is a highly dynamic region of the brain that is continually sensing and responding to changes in the nutrition status of the body. It has been extensively shown that the hypothalamus retains plasticity throughout life, and indeed this is required for hypothalamic function. In addition to the high plasticity in the adult hypothalamus, development of the hypothalamus is closely coupled to the external environment (via the involvement of metabolic hormones in neurodevelopmental processes) and is therefore extremely vulnerable to disruption in both the *in utero* and neonatal period. In this section we describe how exposure to maternal obesity alters hypothalamic structure and function in the offspring (summarised in **Figure 1**). Interestingly, many of the routes through which maternal obesity impacts on the offspring hypothalamus are similar to disruptions observed in human adults with obesity (for e.g.: reduced hypothalamic proliferation (36), disrupted wiring of melanocortin pathways (22), altered nutrient sensing (37)), although in the context of adult obesity it is hard to separate cause and effect.

It is important to note that due to the inherent differences between the sexes in both brain structure and control of metabolism, the consequences of exposure to maternal obesity in the offspring hypothalamus may vary between the sexes. Unfortunately, to date not enough studies have been conducted in both sexes to draw clear conclusions on sex differences in this brain region. However, there is some evidence that the maternal obesity related programming of hypertension and altered heart rate *via* the melanocortin system in the hypothalamus may be sex- specific (38), as may be programming of hypothalamic- pituitary- adrenal axis (39) and glucose-sensitive gene transcription regulation in the PVH (40). For a general review of sex differences in the offspring phenotype following exposure to maternal obesity, see (41).

### 3.1 Cell guidance signals

As discussed above, axon guidance cues such as netrins and semaphorins are essential for correct hypothalamic development. Unfortunately, how these signals may be pathogenically altered is understudied in animal models of maternal obesity. However, a mouse study has shown that the offspring of obese mothers have altered levels of the Netrin receptors Dcc and Unc5d in the fetal ARC, and this is associated with significantly reduced NPY fibre innervation of the PVH compared with that in offspring from lean mothers (42). A recent study by van der Klaauw et al. (22) provides direct evidence that disrupting semaphorin systems in the hypothalamus leads to early-onset obesity in zebrafish, mice, and humans. This research identified 40 rare variants in semaphorin 3 signalling in people living with severe obesity which caused disruption of signalling in melanocortin circuits. This is the first evidence that disruption to the machinery required for axonal guidance in the hypothalamus can cause obesity and highlights the need for more research investigating how this process is altered in the context of maternal obesity.

### 3.2 Neurogenesis

Neurogenesis occurs throughout life in the hypothalamus. The impact of maternal obesity on hypothalamic neurogenesis appears to vary depending on age of the offspring. Maternal obesity results in a reduction in the proliferative potential of hypothalamic neural progenitor cells generated from the fetal hypothalamus on E13 (43) and neurogenic markers in newborn mice (44), whereas an increase in proliferative markers has been reported in the hypothalamus of mice born from an obese pregnancy at PND 21 (45). Whether the early reductions in neurogenesis reflect a permanent reduction, or simply a delay in the peak of proliferation in the embryonic hypothalamus is currently unclear. The Notch signalling pathway is key for the regulation of neurogenesis within the hypothalamus. Exposure to maternal obesity results in an up-regulation of Notch signalling in the neonatal period (43), and a concomitant decrease in expression of the pro-neural transcription factor Mash (44) which may underlie some of the reports of reduced neurogenesis.

### 3.3 Alterations to the melanocortin system

The central melanocortin system is a collection of circuits capable of sensing signals from a wide array of hormones, nutrients and neural inputs. Long-term energy signals from leptin and insulin received by the hypothalamus are integrated with acute signals regulating hunger and satiety, primarily received by the brainstem. Exposure to maternal obesity has been shown to alter frequently studied components of this system are the NPY/AgRP and POMC positive fibres projecting from the ARC to the PVH. These projections are reduced in numerous animal models of maternal obesity and/or diabetes, ranging from rodents to NHP (46–49). Reduction in the number of projections of POMC expressing neurons is also observed in offspring exposed to maternal over-nutrition exclusively during the post-natal period, reflecting the fact that these projections form post-natally in rodents and are vulnerable to disruption during this developmental time window (50). Previous studies have also shown increased gene expression of the target of ARC POMC containing projections- the melanocortin 4 receptor (MC4R) in the PVH- in offspring exposed to maternal obesity, which is also modulated by pup nutrition in the post-natal period (51). A recent study of the translatomic signatures of POMC neurons in offspring after exposure to maternal obesity revealed an altered translatome consistent with other reports of aberrant neuronal development and axonal growth (52).

### 3.4 Nutrient sensing

One of the primary roles of the hypothalamus is to sense changes in nutrient status in the rest of the body, *via* information received from circulating hormones and nutrients. Exposure to maternal obesity is associated with signs of hypothalamic insulin resistance in offspring in both during the *in utero* and post-natal period (43, 53). Many rodent models have utilised post-natal small litter rearing as a way to cause neonatal over-nutrition, and show outcomes similar to the over-nutrition that is experienced in the post-natal period in models of maternal obesity and/or maternal over-nutrition. Neonatal overnutrition is associated with resistance to a host of metabolic hormones including ghrelin (54), leptin (55) and insulin in ARC neurons (56). Studies across a range of species have shown that exposure to over-nutrition- whether *via* maternal HFD consumption or direct induction of hyperglycemia during development- reduces the sensitivity of neurons to glucose in areas of the hypothalamus including the PVH and VMH (40, 57, 58), as well as altering expression of the leptin receptor in the VMH (59). Perhaps due to the inherent sex differences in nutrient sensitivity in the hypothalamus, these programmed effects in offspring are reported to be sex specific (40). Although less studied, there are reports that exposure to maternal obesity also results in altered hypothalamic sensing of fatty acids and their downstream metabolism in the ARC and PVH (Furigo and Dearden, unpublished observation) (57, 60). Furthermore, hypothalamic transcriptome regulation in response to whole body nutrient status (in this example: response to fasting) are altered in offspring of obese mothers in a rat model (61).

Many of the studies mentioned here suggest that the reduced nutrient and hormonal sensitivity is due to altered signalling either at the level of receptor activation, or in downstream pathways (for e.g. in gene expression). However, it should also be noted that disruptions in nutrient sensing may be in part due to altered permeability of the blood brain barrier to circulating peripheral signals, as has been reported in mouse offspring exposed to maternal obesity (62).

### 3.5 Studies of the human hypothalamus in pregnancies complicated by maternal obesity and/or GDM

Due to the lack of tissue for experimental studies, the preceding section is based on observations primarily in rodent models. However, some notable brain imaging studies in the fetuses of women living obesity or GDM have been undertaken in recent years. During pregnancies complicated by maternal obesity, both maternal and fetal insulin levels are high in response to maternal hyperglycemia, and the fetuses of obese mothers develop insulin resistance whilst *in utero* (63). The effects of persistent hyperinsulinemia on brain development are not well characterised, however a recent study in humans has shown that fetal brain activity is altered in response to a maternal oral glucose challenge, and that the magnitude of fetal brain response is correlated with maternal insulin sensitivity (64). Furthermore, fetal postprandial brain responses are slower in the offspring of women with GDM, indicating that GDM directly affects fetal brain activity and may lead to central insulin resistance in the fetus (65). This apparent change in fetal brain glucose and insulin sensitivity is not rescued by late pregnancy lifestyle interventions, suggesting that changes are programmed early in gestation (66). Similarly, a recent MRI imaging study in fetuses from pregnancies complicated by GDM has shown evidence of gliosis in the fetal mediobasal hypothalamus (containing the ARC and other closely surrounding hypothalamic nuclei) that is present early in pregnancy, occurring pre-28 weeks of gestation (67). Due to the observational nature of these imaging studies, it is not possible to draw any conclusions on whether the reported changes in the hypothalamus are causative or indicative of later dysfunction in hypothalamic regulation of energy homeostasis. They do however prove that in human pregnancies, the nutritional state of the mother has a direct impact on the fetal hypothalamus.

## 4 Mechanisms underlying the effects of maternal obesity on offspring hypothalamus

**Figure 2:**
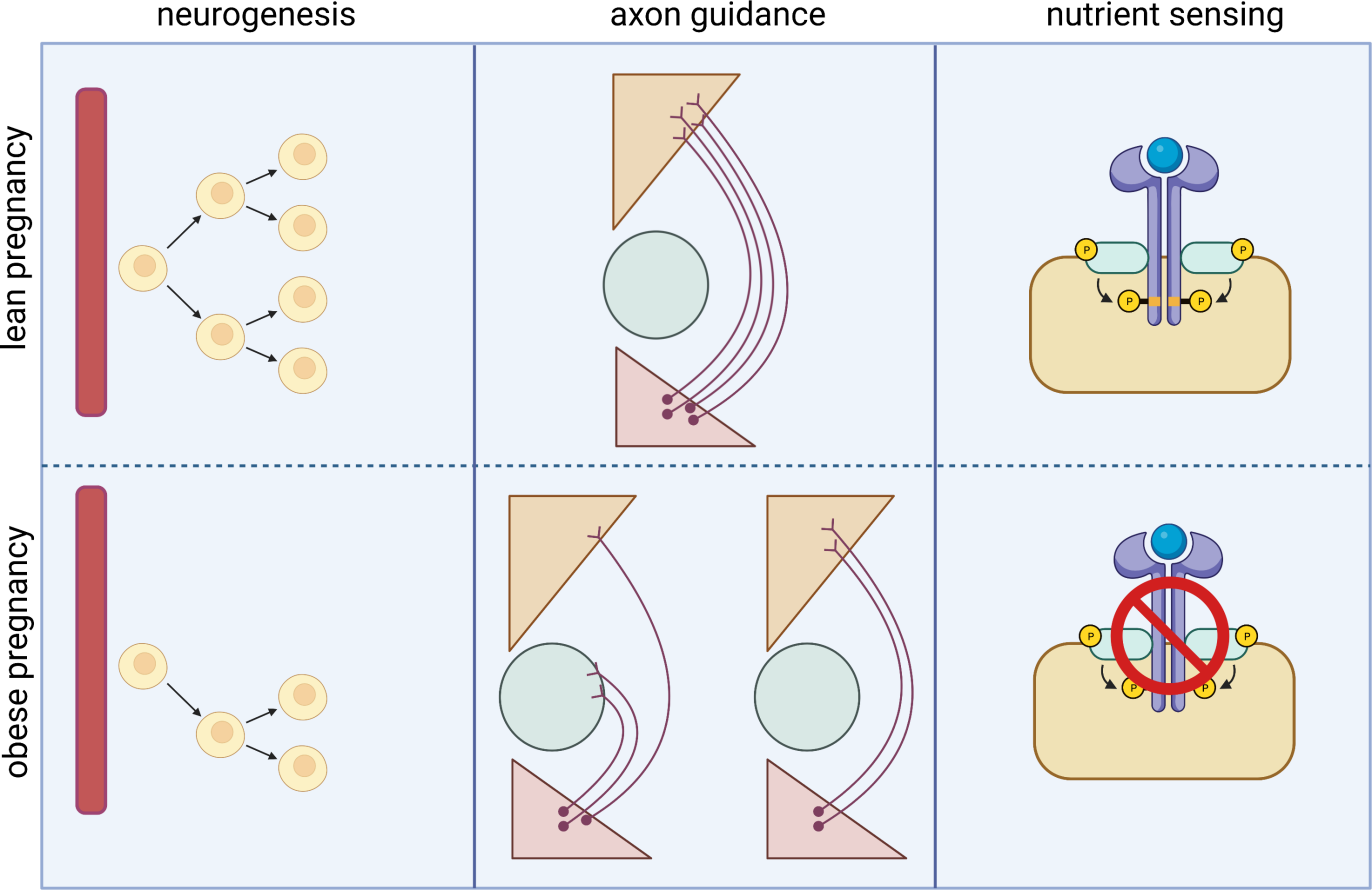
Heat map depicting changes in hormone/nutrient levels across the course of pregnancy in lean and obese mothers. The change in circulating levels of metabolic hormones and nutrients such as glucose and lipids throughout the course of a normal, lean pregnancy are shown in the left panel. Many of these factors are altered in women living with obesity and are thus altered also in pregnancies complicated by obesity with GDM as depicted in the right panel.

There is clearly a strong impact of the peri-natal environment on hypothalamic development, and this is likely to contribute to the increased obesity risk in offspring exposed to maternal obesity. However, the precise mechanisms by which maternal obesity impacts on long-term hypothalamic control of energy homeostasis remain largely undefined. In this section we will discuss the molecular mechanisms with the strongest evidence to date. Pregnancy is a time of high energy demand for the mother, and as such during pregnancy the body makes a series of metabolic adjustments to support the growing fetus. Many metabolic hormones or nutrients are also altered in obesity, and thus remain altered- or are further dysregulated- in a pregnancy complicated by obesity or GDM. As maternal obesity is a major risk factor for GDM, disentangling the effect of maternal obesity *per se* from that of maternal GDM on pregnancy outcomes in women with obesity and diabetes in pregnancy is almost impossible without very large cohorts with refined metabolic measures. Therefore, this section refers to many studies that are complicated by both obesity and GDM, but we have attempted to differentiate between the two where possible. These metabolic changes across pregnancy and in lean or mothers with obesity are summarised in **Figure 2**. Although discussed separately in this section, there is likely considerable overlap between these pathways.

### 4.1 Leptin

The adipokine leptin is released from adipose tissue in proportion to adipose tissue mass. In humans, leptin levels rise throughout pregnancy, reaching a peak in the third trimester. In mothers with overweight or obesity, pre-pregnancy serum leptin levels are already raised, so although they rise less during gestation than in a normal weight pregnancy, they remain higher at term in the mother (68) and fetus (69). The higher leptin levels in an obese pregnancy may contribute to some of the well-known maternal adverse health outcomes: leptin is known to contribute to obesity- related hypertension (70) and leptin concentrations are higher in women with preeclampsia compared with normotensive controls (71, 72) and thus may mediate some of the relationship between higher maternal BMI and preeclampsia risk.

In addition to playing an important role in controlling energy homeostasis, leptin has a significant role in neurodevelopment, particularly in development of the hypothalamic feeding circuitry (73). The formation of POMC and NPY/AgRP positive projections from the ARC to the neuroendocrine and autonomic regions of the PVH in rodents coincides with the timing of the post-natal leptin surge, from around PND 4 to PND 14, in neonatal mice and rats (74). Experiments in rodents manipulating the post-natal leptin surge have shown that not only is the surge necessary for the establishment of these ARC to PVH projections (75), but manipulation of leptin levels in the post-natal period have long-term effects on body weight control. Experimental blockage of the neonatal leptin surge affects gene expression of growth factors, glial proteins, and neuropeptides involved in the control of metabolism and reproduction in peripubertal male and female rats and is associated with increased susceptibility to develop diet-induced obesity (76). Conversely, in a separate mouse model which allowed manipulation of circulating leptin levels during discrete time windows, mice who experienced transient hyperleptinemia from PND0 to PND22 showed a greater susceptibility to develop obesity as adults (77). There studies suggest that the relationship between post-natal leptin and later body weight is a U- shaped curve, and that deviation from normal leptin levels in either direction can increase obesity risk.

Several studies examining hypothalamic architecture in leptin deficient Ob/Ob mice have defined the critical time window when leptin exerts neurotrophic effects within the hypothalamus, but shown that not all projections from the ARC to the PVH are rescued by post-natal leptin replacement in leptin deficient mice (75, 78). A more recent study that analysed the effect of leptin receptor rescue in young (4 weeks) and adult (10 weeks) mice formerly null for the leptin receptor suggests that the development of ARC neural projections can be rescued further into adulthood than previously thought (79). However, a reduction in hypothalamic Pomc, Cartpt and Prlh mRNA expression are persistent in adulthood in this rescue model, which may explain the permanent metabolic alterations caused by early defects in leptin signalling.

The post-natal leptin surge can be altered by the perinatal nutritional environment. Rodent pups reared by obese, or HFD-fed dams have an augmented and prolonged leptin surge magnitude (46, 80). Conversely, the leptin surge is reduced in models of intra- uterine growth restriction (81, 82). Furthermore, mice reared in small litters to induce post-natal over-nutrition display an augmented plasma leptin surge, whereas large litter size- a model of under-nutrition- is associated with a delayed surge of reduced magnitude (80). Manipulation of leptin levels may be a route to overcome programmed effects due to nutrition in the perinatal period. Collden et al. have recently shown that neonatal administration of a leptin antagonist normalises adiposity and hypothalamic leptin sensitivity in postnatally over-nourished mice (55). Taken together, these studies have shown that leptin is an important trophic factor for the development of hypothalamic feeding circuits critical for the control of energy balance, and that altered leptin levels are a likely route by which the nutritional environment in the peri-natal period alters energy homeostasis control.

### 4.2 Insulin

At the start of pregnancy, there is an initial rise in insulin secretin and sensitivity in the mother which stimulates lipogenesis and reduces fatty acid oxidation, causing maternal fat storage. Around mid-gestation, insulin resistance develops to direct all available fuel towards the growing fetus. This natural state of insulin resistance during pregnancy is worsened in pregnancies complicated by GDM or obesity (83–85). Interestingly, the augmented insulin resistance seen in GDM pregnancies seems to be caused by enhanced activity of the same mechanisms present in an uncomplicated pregnancy, rather than *via* novel pathogenic routes (86). Enhanced maternal insulin resistance in obese and GDM pregnancies contributes to high maternal glucose levels, leading to a state of hyperglycemia in the mother and fetus, since glucose freely crosses the placenta. This then leads to a compensatory rise in fetal insulin levels (63). Women living with obesity even without GDM have higher glucose profiles on continuous glucose monitoring performed during pregnancy than normal weight women (87). Maternal glycemia is a strong determinant of fetal growth, as demonstrated by the strong, continuous associations of maternal glucose levels with increasing birth weight (88).

It has long been established that insulin plays a neurotrophic role in numerous brain regions and across a range of species (89–93). Insulin also has an important function in both embryonic and adult stem cell homeostasis *via* a role in maintaining neural stem cell self-renewal, neurogenesis and, in some instances, promoting differentiation (94, 95). Insulin also acts to inhibit neuronal apoptosis *via* activation of protein kinase B and protein kinase C (96, 97) resulting in increased neuronal survival. Artificial manipulation of insulin in the brain during the perinatal period *via* the implantation of insulin containing agar implants results in an altered ratio of neuronal to glial cells in the VMH (98).

There is also evidence from rodent studies that insulin signalling is required both in the pre- and post- natal periods for correct development of hypothalamic projections. Although the genetic deletion of InsR from POMC neurons does alter their development under normal conditions, it prevents the reduction of ARC POMC projections to the pre-autonomic compartment of the PVH that occurs in offspring exposed to maternal over-nutrition, suggesting this particular disrupted circuit development in response to maternal nutrition is mediated through insulin signalling (50). It has recently been shown that the impact of insulin on growth of primary neuronal cultures originating from the ARC is dependent on the nutrient availability in the postnatal period, further demonstrating an important interaction between insulin signalling and nutritional state in determining neuronal growth and circuit formation (99).

High insulin levels and fetal brain insulin signalling are essential for appropriate brain maturation. However, chronic hyperinsulinemia, which is present in insulin resistant mothers and corresponds to high insulin levels in the fetus, can induce insulin resistance in the fetus (63). We have previously reported that the fetuses of obese, hyperinsulinemic mice display reduced expression of proliferative genes in the hypothalamus and disrupted neural stem cell growth in primary culture, and that these two markers of neuronal proliferation were correlated with maternal insulin levels (43). Due to the essential role for insulin signalling in neural stem cell self-renewal and neurogenesis, insulin resistance in the developing hypothalamus could explain the reduced proliferative response of hypothalamic neurons in offspring exposed to an environment of energy excess (43, 56) that results in long term morphological changes in the hypothalamus and ultimately a lack of energy balance regulation.

### 4.3 Ghrelin

Ghrelin is a gut hormone with a strong orexigenic signal. Following release into the circulation, ghrelin circulates as two major forms: acyl- ghrelin and desacyl- ghrelin. Maternal total ghrelin concentrations decrease slightly throughout pregnancy, and there is a positive correlation between the ratio of acylated to total circulating ghrelin in the mother during the third trimester of gestation and infant birth weight (100). Circulating maternal desacyl- ghrelin is increased in pregnancies with GDM, possibly reflecting resistance to the inhibitory effect of insulin on ghrelin secretion (100). Cord blood total ghrelin levels are inversely correlated with birth weight and are decreased in women with GDM (101) concomitant with the increased birth weight seen in babies from GDM pregnancies.

Rodent and human studies have suggested that maternal ghrelin regulates fetal development during the late stages of pregnancy. Administration of ghrelin to mice during the last week of gestation causes a 10–20% increase in offspring birth weight (102). This effect is persistent even when maternal food intake after ghrelin treatment is restricted through paired feeding, suggesting a direct action of ghrelin on the fetus trough a transplacental transfer. Importantly, studies performed in mice to block ghrelin action during early post-natal development have shown an enhancement of ARC neural projections that are associated with long-term metabolic effects (103). It appears that ghrelin plays an inhibitory role in the development of hypothalamic neural circuits- acting as the “break” in balance to the neurotrophic action of leptin- and therefore correct expression of ghrelin, similar to leptin, during neonatal life could be important for later hypothalamic regulation of energy homeostasis.

### 4.4 Fatty acids and links to endoplasmic reticulum stress/inflammation

During pregnancy there is an accumulation of lipids in the first and second trimester in the mother and later increased lipolysis of maternal adipose tissue stores. The catabolic state of maternal adipose tissue during late gestation is associated with hyperlipidemia, mainly corresponding to plasma rises in triglycerides, with smaller rises in phospholipids and cholesterol (104). There are conflicting reports as to whether maternal cholesterol levels are altered in a pregnancy complicated by GDM (104). Maternal obesity is however associated with an increase in maternal lipid levels, higher triglycerides and VLDL, and lower HDL-C than observed in lean women (105). GDM in women with obesity is associated with elevated plasma concentrations of a specific series of triglycerides consistent with increased *de novo* lipogenesis (106). Several recent studies indicate that maternal post-prandial triglycerides and free fatty acids are a stronger predictor of newborn adiposity and birth weight than maternal glucose in an obese pregnancy (106, 107).

The quantity and specification of fatty acids ingested by the mother during pregnancy is of importance for brain development and hypothalamic function in the offspring. Hypothalamic dysfunction is observed in mice and rat offspring born to mothers that ingested increased amounts of saturated or trans fatty acids (108, 109). Park et al. (47) have demonstrated that exposure to high concentrations of a specific combination of fatty acids (designed to mimic the commonly used high- fat diets in diet- induced obesity studies) causes reduced neurite outgrowth from the ARC, suggesting that this may be a cause of the widely reported phenotype of reduced ARC to PVH projections in the offspring of obese mothers.

Endoplasmic reticulum (ER) stress and inflammation are important mechanisms that link changes in fatty acid levels to hypothalamic impairment. Over-nutrition typically activates hypothalamic inflammatory signalling at least in part through elevated ER stress in the hypothalamus and this might be a general neural mechanism for energy imbalance underlying obesity (110, 111). In the context of an obese pregnancy, ER stress and inflammation are increased in the offspring hypothalamus (53) and the former is reported to be a consequence of elevated circulating fatty acids in obese dams and their offspring (47). Furthermore, neonatal treatment with tauroursodeoxycholic acid, an ER stress-relieving drug, ameliorates the metabolic and neurodevelopmental deficits observed in these animals, suggesting the increase in ER stress is causative of later metabolic dysfunction (47). Therefore, it is suggested that fatty acids play an important role in the hypothalamic dysfunction observed in offspring born to mothers that ingest increased amounts of saturated or trans fatty acids, and the mechanisms underlying these alterations may be through ER stress and hypothalamic inflammation.

### 4.5 Growth hormone

Growth hormone (GH) is well known for its function to stimulate growth, cell reproduction, and cell regeneration, and as such is hugely important for development. A growing body of evidence has shown the brain is an important target of GH for the regulation of food intake, energy expenditure, and glycemia, particularly in response to different forms of metabolic stress such as glucoprivation, food restriction, and exercise (112–114). During pregnancy, central GH action is related to the regulation of food intake, fat retention, and sensitivity to insulin and leptin in the mother, suggesting that GH acts in concert with other gestational hormones to prepare the maternal organism for the metabolic demands of the offspring (115). Interestingly offspring born to mothers with a genetic knockout of the GH receptor in cells that co-express leptin receptor exhibit a significantly lower growth rate from the second week of life, compared to offspring born to mothers with a loss of GH receptor in the entire brain or wild type mothers (115).

Despite the importance of GH in fetal development, little is known about the programming effects of maternal and/or fetal GH on offspring hypothalamic development. There is some evidence that GH regulates hypothalamic neurocircuits controlling energy homeostasis from experiments demonstrating a direct trophic role of GH on the formation of POMC and AgRP axonal projections (116). Mice with deletions of GH receptor in neurons that co-express leptin receptor have decreased density of POMC positive neuronal innervation in the PVH and DMH. Conversely, AgRP-specific ablation of GH receptor led to a significant reduction in AgRP projections to the PVH, LHA and DMH, without affecting POMC innervation (116). Further studies are needed to define whether altered maternal or fetal GH levels have other impacts on the developing hypothalamus, and whether changes are causative of later obesity.

## 5 Conclusions and future perspectives

Due to the increasing number of women who are overweight or living with obesity before entering pregnancy, it is essential that we investigate the long-term consequences of this exposure for their offspring. A greater understanding of the mechanisms by which the maternal environment acts on the offspring hypothalamus to disrupt energy homeostasis control is essential to reduce the inter-generational transmission of obesity risk. It is becoming increasingly understood that people living with obesity are fighting against their biology to maintain a healthy body weight, and the developmental programming of obesity risk is an underappreciated factor in this issue that determines our lifelong health before we are born. Whilst there are undoubtedly many contributing factors to the growing obesity epidemic, interventions in pregnancies complicated by obesity give us a unique opportunity to improve the health of two generations at the same time, and at a time when an individual is in frequent contact with health professionals. Future research in this field should aim to fill the gaps in our knowledge that will allow us to provide detailed information to enable mothers to make informed choices for their unborn offspring.

## Author contributions

IF wrote the manuscript, LD conceived ideas, made figures and wrote the manuscript. All authors contributed to the article and approved the submitted version.
